# Morphine electrochemical determination using SnO_2_ nanostructure-modified glassy carbon electrode in the presence of diclofenac

**DOI:** 10.5599/admet.2803

**Published:** 2025-07-02

**Authors:** Zainab S. Hadawi, Isam Ngaimesh Taeb, Rasha N. Aljabery

**Affiliations:** 1 Department of Chemistry, College of Science, University of Thi-Qar, Thi-Qar, 64001, Iraq; 2 Pathological Analyses Department, College of Science, University of Sumer, Iraq

**Keywords:** Electrochemical sensor, voltammetric determination, modified electrodes, voltammetry, chronoamperometry

## Abstract

In the present work, SnO_2_ nanostructures were synthesized and a sensitive voltammetric sensor on a glassy carbon electrode (GCE) was constructed to estimate morphine (MP) in the presence of diclofenac (DLF).

**Background and purpose:**

Because diclofenac (DLF) is an NSAID, its administration can reduce postoperative morphine (MP) requirements in adults; for example, standard DLF dosing has been shown to decrease MP use after abdominal surgery. Hence, devising a simple, cost-effective, and swift assay for these compounds in biological and pharmaceutical specimens is indispensable.

**Experimental approach:**

SnO_2_ nanostructures were synthesized, and a sensitive voltammetric sensor on a glassy carbon electrode (GCE) was constructed to estimate MP in the presence of DLF. Cyclic voltammetry was employed to evaluate the electrochemical response of the SnO2 nanostructures/GCE towards MP.

**Key results:**

The SnO_2_ nanostructures exhibited a significant effect on the electrochemical reaction of the electrode toward the MP oxidation. The SnO_2_ nanostructures/GCE further exhibited a more sensitive detection platform for MP determination with a limit of detection of 0.006 μM using differential pulse voltammetry in a linear range of 0.01 to 340.0 μM.

**Conclusion:**

The SnO_2_ nanostructures/GCE exhibited extremely high electrochemical activities towards the simultaneous oxidation of MP and DLF. Moreover, the SnO_2_ nanostructures/GCE provided reproducible and stable responses for MP quantitation. The platform prepared showed successful performance for MP and DLF determination in real samples. SnO_2_ nanostructures exhibited a significant effect on the electrochemical reaction of the electrode toward the MP oxidation. The SnO_2_ nanostructures/GCE further exhibited a more sensitive detection platform for MP determination with a limit of detection of 0.006 μM using differential pulse voltammetry in a linear range of 0.01 to 340.0 μM. Additionally, the SnO_2_ nanostructures/GCE exhibited extremely high electrochemical activities towards the simultaneous oxidation of MP and DLF. Moreover, the SnO_2_ nanostructures/GCE provided reproducible and stable responses for MP quantitation. The platform prepared showed successful performance for MP and DLF determination in real samples.

## Introduction

**Figure 1.**
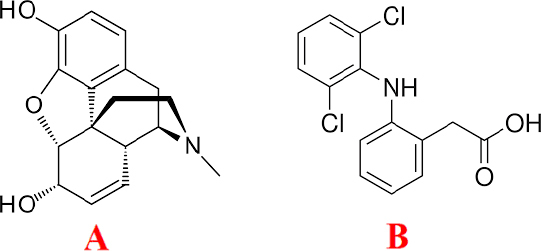
Chemical structure of MP (A) and DLF (B).

Morphine (MP) (Figure 1A) is considered to be the gold standard or analgesic standard for treating severe or painful pain and suffering [1]. MP, however, is banned for use in sports doping.

A benefit of MP use would be the alleviation of pain during a grueling athletic competition, pain that could complicate performance. Research indicates that if a sportsperson is administered MP during training (which is permissible), then does not have it long enough for the MP to have left the body and then receives a saline injection (placebo) on competition day, the sportsperson experiences less pain [2]. A rapid, highly sensitive, and selective method for measuring MP in pharmaceutical and biological matrices is therefore essential. Various analytical techniques have been applied to MP quantification, including high-performance liquid chromategraphy [3], spectrophotometry [4], gas chromatography–mass spectrometry [5], fluorimetry [6], surface plasmon resonance (SPR) [7], chemiluminescence [8], and several electroanalytical approaches [9,10].

Diclofenac (DLF) (Figure 1B) is widely regarded as both safe and effective for treating a range of inflammatory and rheumatoid conditions [11]. Following oral administration, DLF is absorbed efficiently and undergoes extensive hepatic metabolism. It exhibits a terminal half-life of 1–2 hours, a volume of distribution of 0.17 L/kg, binds 99 % to plasma proteins, and penetrates the synovial fluids [12]. Accurately detecting minute quantities of DLF in pharmaceutical formulations is critical for pharmaceutical quality control and therapeutic monitoring. Accordingly, there is a pressing need for an analytical technique that is straightforward, selective, and rapid, economical for trace-level determinations of DLF across various drug preparations. Moreover, because DLF is an NSAID, its administration can reduce postoperative MP requirements in adults—for example, standard DLF dosing has been shown to decrease MP use after abdominal surgery [13]. Hence, devising a simple, cost-effective, and swift assay for these compounds in biological and pharmaceutical specimens is indispensable. The glassy carbon electrode (GCE) is a preferred substrate in electrochemistry due to its wide potential window and minimal background current. However, its intrinsic slow surface electron-transfer kinetics limit performance. To overcome these shortcomings, researchers often employ modified electrodes [14].

Electrode modification is particularly valuable because it lowers overpotentials and enhances electron-transfer rates compared to unmodified surfaces, thereby boosting sensitivity and selectivity in detecting bioactive drugs and biomolecules [15].

Nanomaterials have garnered significant attention across numerous fields owing to their distinctive characteristics and versatile applications. In analytical chemistry, nanostructured materials are prized for their remarkable stability, catalytic efficiency, and high surface area [16]. When incorporated into electrochemical sensors as nanocomposites, they markedly improve the precision and sensitivity of analyses in biological and pharmaceutical contexts. Thanks to their diverse sizes, morphologies, and compositions, nanocomposites are reshaping analytical methodologies [17].

Recently, SnO_2_ nanostructures have emerged as a focus of research because they combine cost-effective synthesis with high thermal stability, biocompatibility, chemical and photochemical inertness, a wide band gap, and superior facilitation of electron transfer in redox processes [18]. Accordingly, SnO_2_-based nanomaterials—exhibiting similar advantageous properties—have found widespread utility in a variety of applications [19].

In this work, we synthesized SnO_2_ nanosheets and employed them to modify a GCE surface. We characterized the modified electrode using cyclic voltammetry (CV) and demonstrated its application as a novel electrocatalytic platform for MP detection in phosphate buffer. We further evaluated the analytical capabilities of the SnO_2_ nanosheet–modified GCE for the simultaneous quantification of MP and DLF, successfully applying the method to real sample analysis.

## Experimental

### Apparatus and reagents

Every electrochemical experiment was performed with a potentiostat/galvanostat μAutolab Type III (Eco Chemie B. V., The Switzerland). A three-electrode cell was employed. Platinum wire, SnO_2_-modified GCE, and Ag/AgCl (KCl, Sat.) electrodes were used as auxiliary working and reference electrodes, respectively. The reagents were analytical grade (Merck), including MP and DLF. Orthophosphoric acid and its salt combinations with pH between 3.0 and 9.0 were used to create phosphate buffers (0.1 M).

### Synthesis of SnO_2_ nanostructures

**Figure 2.**
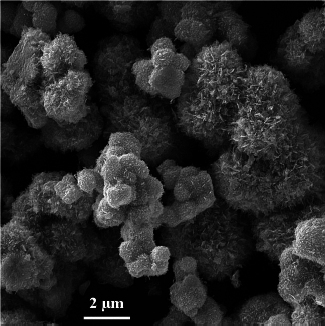
FESEM image of SnO_2_ nanostructures

With minor modifications, SnO_2_ nanostructures were synthesized using a solvothermal/hydrothermal method based on Li et al.'s methodology [20]. First, 20 mL of ethanol and 15 mL of deionized water were used to dissolve 5 mmol of SnCl_2_·2H_2_O (1.128 g). 5 mL of 0.5 M NaOH solution was added dropwise while being stirred magnetically. After 5 minutes of stirring with 0.5 g of polyvinylpyrrolidone (PVP), 10 mmol of trisodium citrate dihydrate (2.941 g) was added, and the mixture was agitated for another 50 minutes. After that, the resultant precursor solution was moved to an autoclave lined with Teflon and heated for 12 hours at 180 °C in a water bath. After the reaction in the autoclave was finished, it was taken out and allowed to cool naturally to room temperature. Centrifugation was used to collect the precipitates, which were then repeatedly cleaned with deionized water and ethanol before being dried for 15 hours at 67 °C in an oven. The dry powder was then calcined for two hours at 500 °C. A typical FESEM image can be seen in Figure 2.

### Modification of glassy carbon electrode with SnO_2_ nanostructures

The bare GCE was first polished using alumina slurries on a polishing pad, then completely washed with deionized water and allowed to air dry before being modified. To create a homogenous solution, one milligram of the produced SnO_2_ nanostructures was dissolved in 1.0 milliliter of deionized water and sonicated for twenty minutes. The SnO_2_ nanostructures-coated GCE (SnO_2_/GCE) was then prepared by drop-casting a 3 μL aliquot of this solution onto the GCE surfaces and letting it cure at room temperature. Both the modified and unmodified electrodes' electrochemically active surface area (EASA) was measured using cyclic voltammetry at different scan speeds in 0.1 M KCl with 1.0 mM K_3_[Fe(CN)_6_]. The EASA of SnO_2_/GCE was determined to be 0.128 cm^2^ using the Randles–Ševčík equation, which is around 4.1 times higher than that of the unmodified GCE.

### Preparations of a real sample

Healthy participants provided urine samples, which were promptly chilled. After centrifuging each urine sample for 15 minutes at 2000 rpm, the clear supernatants were filtered through a 0.45 μm membrane. Using PBS (pH 7.0), the filtrate was diluted to 25 mL volume in volumetric flasks containing the appropriate quantities. The standard addition method was used to measure the quantities of MP and DLF added to these diluted urine samples at established concentrations.

0.1 M PBS (pH 7.0) was used to dilute 1 mL of a 10 mg/mL ampoule to 10 mL in order to prepare the MP stock. After that, aliquots of this solution were put into different 25 mL flasks and filled with PBS. Standard additions were used to determine the concentration of MP and DLF in these solutions. Five 50 mg DLF pills were ground into a fine powder for analysis. Using ultrasonication, 150 mg of this powder was dissolved in 25 mL of water. In a 25 mL volumetric flask, the resultant solution was further diluted using PBS (pH 7.0). The conventional addition procedure was also used to quantify MP and DLF.

## Results and discussion

### Electrochemical response of morphine on various electrodes

We examined how MP’s oxidation behaved across a pH range from 4.0 to 9.0 in 0.1 M PBS to assess the influence of proton concentration on its electrochemical signal. The peak oxidation current of MP increased with a pH increase, achieving its highest value at pH 7.0, before declining at more alkaline conditions. Therefore, pH 7.0 was selected for all subsequent electrochemical experiments.

**Figure 3.**
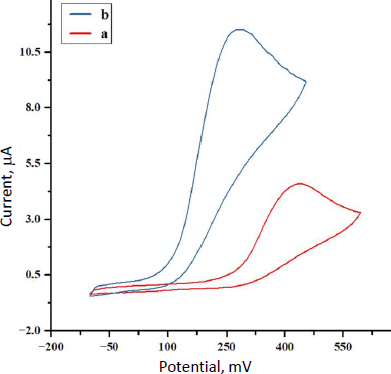
CVs of 150.0 μM MP recorded at a scan rate of 50 mV s^-1^ for (a) bare GCE and (b) SnO_2_ nanostructures /GCE

Cyclic voltammetry was then employed to compare MP oxidation at (a) an unmodified GCE and (b) the SnO_2_ nanostructures-modified GCE. As shown in Figure 3, at pH 7.0 with 150.0 μM MP, the bare GCE produced a small, broad oxidation peak of 4.6 μA at +440 mV. In contrast, the SnO_2_/GCE electrode exhibited a negative shift in peak potential (160 mV) along with a higher peak current of 11.5 μA. This significant increase in current and shift toward lower oxidation potential indicate that the SnO_2_ nanostructures greatly catalyse MP oxidation.

### Effect of scan rate

**Figure 4.**
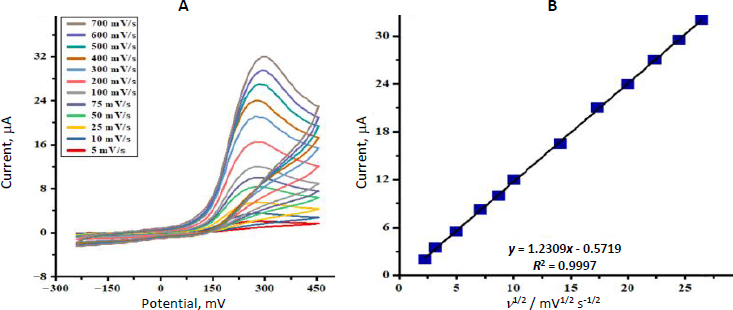
(A) CVs recorded for the MP (100.0 μM) oxidation on the SnO_2_ nanostructures/GCE at different scan rates (B) Plot of the peak currents obtained from CVs in Fig. 4A *vs.* the square root of scan rate

The CVs were obtained between the MP (100.0 μM) oxidation at the SnO_2_ nanostructures /GCE at the varying scan rates (*ν*) (Figure 4A). The increase in the oxidation peak with the scan rate was observed between 5 and 700 mV s^-1^. Plotting current (*I*) against *ν*^1/2^ resulted in a linear plot (Figure 4B), which indicates that MP oxidation is controlled mainly by diffusion-controlled kinetics.

### Chronoamperometric analysis

**Figure 5.**
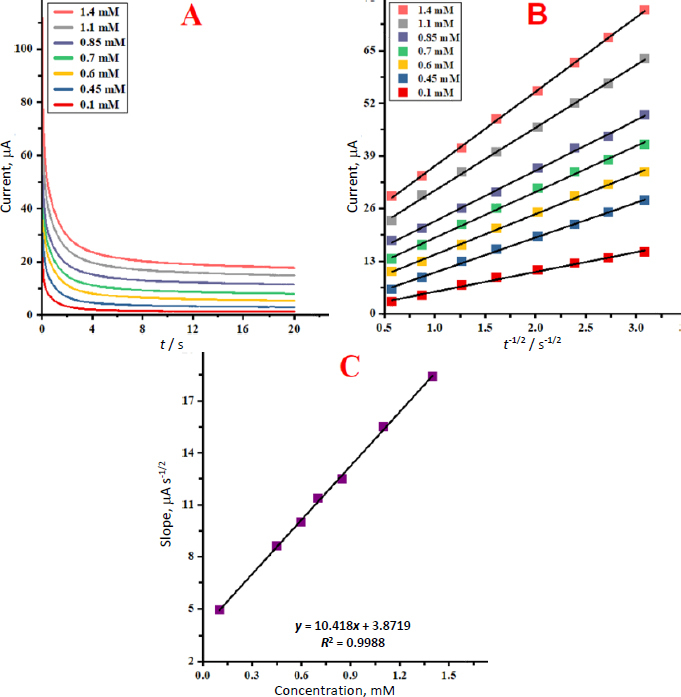
(A) SnO_2_ nanostructures/GCE chromatoamperometric response for varying MP levels. (B) *I vs. t*^−1/2^ plots; (C) plot of the slopes of the straight lines *vs.* MP concentration

Chronoamperometry was employed to examine the MP catalytic oxidations on the SnO_2_ nanostructures/GCE surface. Chronoamperometric measurements for different MP content were performed on SnO_2_ nanostructures/GCE at 350 mV. The chronoamperogram of different MP concentrations on SnO_2_ nanostructures/GCE are displayed in Figure 5A. The Cottrell equation determines the current (*I*) of an electrochemical reaction involving electroactive materials with a diffusion coefficient (*D*) under mass transport-limited conditions. Figure 5B illustrates a linear relationship between the *I* and *t*^-1/2^ for the oxidations of different MP concentrations. The gradients of the generated straight lines plotted against different concentrations of MP are displayed in Figure 5C. The *D* value for MP was determined to be 9.2910^-6^ cm^2^/s using the Cottrell equation and the slope.

### Quantitative determination of MP by differential pulse voltammetry

**Figure 6.**
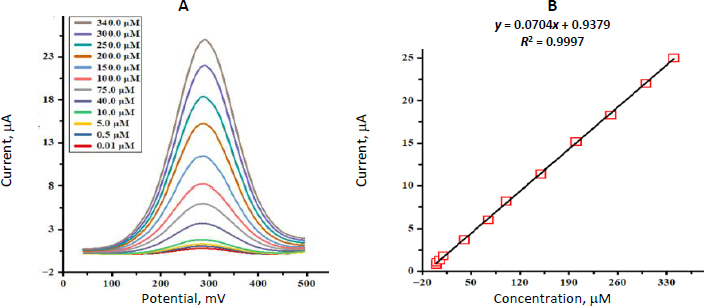
(A) DPVs of the SnO_2_ nanostructures /GCE for increasing MP levels. (B) calibration line of oxidation peak current versus MP concentration

The SnO_2_ nanostructures-modified GCE was employed in the measurements of MP concentrations by a differential pulse voltammetry (DPV) method. DPV recordings for MP from 0.01 to 340.0 μM are presented in Figure 6A. As MP concentration increased, the oxidation peak currents increased correspondingly, indicating the sensor’s excellent responsiveness. A calibration graph plotting peak current against MP concentration exhibited a straight-line relationship over the 0.01 to 340.0 μM interval (6B). The method’s detection limit was determined to be 0.006 μM (*S*/*N* = 3).

### DPV analysis for the determination of MP in the presence of DLF

The electrochemical response of the supplied analytes was determined by altering the concentration of both analytes concurrently to assess the potential of the SnO_2_ nanostructures/GCE to codetect MP in the presence of DLF.

**Figure 7.**
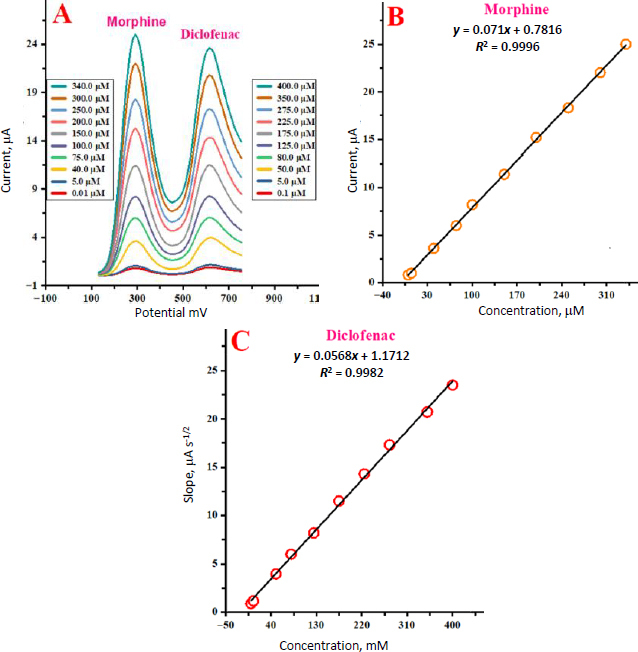
(A) DPV profiles for simultaneous determination of MP and DLF at SnO_2_ nanostructures/GCE. (B) peak current vs. MP concentration; (C) peak current *vs.* DLF concentration

Two non-interference peaks on DPV curves were simultaneously formed by the change of their concentrations, as shown in Figure 7A.

According to Figures 7B and 7C, the peak currents of MP and DLF oxidation increased linearly with the increase in their respective concentrations. The capacity to detect MP and DLF in the mixed solution was demonstrated by the maximum current intensities and excellent linearity with changes in the target concentration.

### Interference study

The specificity of the SnO_2_ nanostructures–coated GCE toward 50.0 μM MP was tested against a variety of potential interfering species. For each substance, the highest concentration that produced less than a ±5 % change in the MP oxidation signal was determined. Even when present at 480 times the MP level, NH_4_^+^, Cl^-^, Mg^2+^, Na^+^, Br^-^, glucose, fructose, and lactose caused negligible interference; histidine, phenylalanine, methionine, alanine, glycine, ethanol, methanol, and tryptophan at 300-fold excess likewise had no effect; ascorbic acid (post-treatment with ascorbic oxidases), tyrosine, thiourea, acetaminophen, and cysteine showed no significant signal change even at 230-fold excess; and dopamine, uric acid, or methyldopa in 10-fold surplus did not alter the response. These findings confirm the excellent selectivity of the SnO_2_/GCE for MP detection.

### Real sample analysis

The proposed approach was also used to determine MP and DLF in MP injection and DLF tablets to assess its analytical application. The samples were created to measure the concentration of MP and DLF acid in pharmaceutical items based on the DPV response (*n* = number of spiked samples with known concentrations of MP and DLF acid and diluted analytes. Table 1 presents the obtained results. By contrasting the results with those listed on the medicinal product label, the suggested modified electrode reliability was further validated (Table 2). The relative standard deviations (RSD) and recovery percentages of the spiked samples are adequate, according to the data shown in Table 1. Furthermore, Table 2's results demonstrate that the values acquired using SnO_2_ nanosheets and GCE are consistent with those listed on the preparations' labels. As a result, the modified electrode may be used to determine MP and DLF in medicinal formulations either separately or together.

**Table 1.** Determinations of MP and DLF in real pharmaceutical preparations using the SnO_2_ nanostructures /GCE, (*n* = 5)

Sample	Amount, μM				Recovery, %		RSD, %	
	Spiked		Found					
	MP	DLF	MP	DLF	MP	DLF	MP	DLF
MP injection	0	0	4.9	-	-	-	3.1	
	1.0	4.0	6.1	3.9	103.4	97.5	1.9	3.3
	3.0	6.0	7.8	6.1	98.7	101.7	2.4	2.8
DLF tablet	0	0	-	3.9	-	-	-	2.1
	4.5	1.0	4.6	4.8	102.2	98.0	2.3	3.1
	6.5	3.0	6.4	7.0	98.5	101.4	2.9	2.5

**Table 2:** Comparisons of the totals value of MP and DLF of some pharmaceutical preparations obtained using SnO_2_ nanostructures /GCE with declared value in the labels of the sample (*n* = 5)

Samples	Declared value	Found value	RSD, %
Content of MT in MP injection, mg ml^-1^	10.0	10.105	2.46
Content of DLF in DLF tablet, mg per tablet	50.0	49.85	2.57

The developed approach was also used to determine MP and DLF in real urine samples in order to verify its analytical validity. Table 3 shows the performance of determination for two species in real samples. MP and DLF experimental values were successfully recovered. The mean relative standard deviation (RSD) was used to verify the repeatability of the procedure.

**Table 3.** Determination of MP and DLF in urine sample using the SnO_2_ nanostructures /GCE. (*n* = 5)

Amount, μM				Recovery, %		RSD, %	
Spiked		Found					
MP	DLF	MP	DLF	MP	DLF	MP	DLF
0	0	-	-	-	-	-	-
4.0	4.5	4.1	4.4	102.5	97.8	3.0	2.2
6.0	6.5	5.9	6.6	98.3	101.5	2.3	3.4

## Conclusions

We have investigated the electrochemical detection of MP and DLF at a glassy carbon electrode modified with SnO_2_ nanostructures. Compared to the unmodified GCE, the SnO_2_-coated electrode exhibited markedly enhanced sensitivity, a lower detection limit, straightforward fabrication, and excellent reproducibility and stability for the simultaneous quantification of both compounds. Notably, the oxidation peak potential of MP shifted from +440 mV at the bare GCE to a less positive value on the SnO_2_/GCE, illustrating the pronounced catalytic effect of the nanostructures modification.
